# The predictive validity of the *Living Goods* selection tools for community health workers in Kenya: cohort study

**DOI:** 10.1186/s12913-018-3620-x

**Published:** 2018-10-20

**Authors:** Celia A. Taylor, Richard J. Lilford, Emily Wroe, Frances Griffiths, Ruth Ngechu

**Affiliations:** 1 Division of Health Sciences, Warwick Medical School, University of Warwick, Coventry, CV4 7AL UK; 2 Partners In Health, Neno, Malawi; 3 Living Goods, PO Box 30261-00100, Nairobi, Kenya

**Keywords:** Community health workers, Selection, Assessment, Performance, Predictive validity

## Abstract

**Background:**

Ensuring that selection processes for Community Health Workers (CHWs) are effective is important due to the scale and scope of modern CHW programmes. However they are relatively understudied. While community involvement in selection should never be eliminated entirely, there are other complementary methods that could be used to help identify those most likely to be high-performing CHWs. This study evaluated the predictive validity of three written tests and two individual sections of a one-to-one interview used for selection into CHW posts in eight areas of Kenya.

**Methods:**

A cohort study of CHWs working for *Living Goods* in eight local areas of Kenya was undertaken. Data on the selection scores, post-training assessment scores and subsequent on-the-job performance (number of household and pregnancy registrations, number of child assessments, proportion of on-time follow-ups and value of goods sold) were obtained for 547 CHWs. Kendall’s tau-b correlations between each selection score and performance outcome were calculated.

**Results:**

None of the correlations between selection scores and outcomes reached the 0.3 threshold of an “adequate” predictor of performance. Correlations were higher for the written components of the selection process compared to the interview components, with some small negative correlations found for the latter.

**Conclusions:**

If the measures of performance included in this study are considered critical, then further work to develop the CHW selection tools is required. This could include modifying the content of both tools or increasing the length of the written tests to make them more reliable, for if a test is not reliable then it cannot be valid. Other important outcomes not included in this study are retention in post and quality of care. Other CHW programme providers should consider evaluating their own selection tools in partnership with research teams.

## Background

There is growing evidence that Community Health Workers (CHWs) can contribute effectively to addressing preventable burdens of disease, particularly by undertaking specific activities such as surveillance for tuberculosis [1] and case detection and treatment for febrile illness in children [2]. This positive evidence, together with task-shifting recommendations to help to alleviate human resources for health crises [3] has led to an increase in the scale and scope of CHW programmes. In terms of scale, a recent modelling study estimated a need for 1 million CHWs to serve the rural population of sub-Saharan Africa [4], while in terms of scope, integrated Community Case Management [5] and other comprehensive models including multiple health domains are replacing disease/activity-specific models [6]. CHW programmes are also becoming more formalised, for example by payment of salaries, compulsory training and monitoring activities [5]. An effective CHW programme requires strong design features such as training, supervision and incentives [7] and CHWs with the knowledge, skills and attributes required to do their jobs well, such as time-management, respect, kindness, empathy and diligence [8, 9]. Some of these competencies are incorporated into CHW training, but others, such as honesty, are less “trainable”. In addition, the ability of CHWs to assimilate the knowledge and skills included in the training curriculum will depend on their personal “trainability”. As a result, levels of knowledge, skill and attributes vary between CHWs and there is evidence that some struggle to cope with the workload [10], leading to poor retention [11] and sub-optimal effectiveness [7, 8].

One design feature that has not received much attention to date is the selection of CHWs [12–14]. Selection is important because an effective selection process would help identify the most “trainable” CHWs and those with the highest levels of the skills and attributes that are less trainable [15]. Such a selection process may also help potential CHWs make an informed decision about their career path by increasing their knowledge of the role, as the selection process may include a briefing about the role and/or introduce applicants to the nature of the role through the scenarios included in the selection assessments. In turn, this should improve retention, as potential CHWs will have a better idea of what the job will involve in practice. K’Omudho & Tenambergen suggest that the lack of attention on selection by programme providers could be one reason for differential CHW performance across programmes: “the absence of standardized procedures for CHW selection affects programme results in different parts of the world.” [13], p. 2). A further explanation for the importance of CHW selection is implied by Nkonki and colleagues, who note that “selection criteria for entry to a Lay Health Worker programme determine the profile of workers it employs” [16], p. 921.

In the traditional, single-disease CHW model, CHWs were both from and selected by their own communities [17]. Given the nature of the CHW role, community engagement in selection should never be eliminated entirely [18] and has been found to be the preferred approach to selection by CHWs themselves in one study [19]. The rationale for community involvement is that communities should be the most knowledgeable about who would be appropriate and which individuals have the necessary relationships. Perhaps most importantly in terms of this rationale, community members have a “right” to be involved [19, 20]. A systematic review has identified that such community involvement may enhance motivation and self-esteem [7] and potentially retention [19], although there are no empirical data available to test this hypothesis. However, relying on community selection alone may not guarantee that the ‘best’ CHWs are selected [21, 22] or that the process is fair [13, 22, 23]. For example, almost half of CHWs included in a 1989 UNICEF survey were related to the village chief or sub-chief; while in Swaziland local chiefs were found to select CHWs based on their own interests, not the qualifications of potential CHWs [18]. Turinawe [23], meanwhile, reports that the selection process in Luwero district of Uganda was so dominated by local village council leaders at the expense of full community participation that CHWs were distrusted, leading them to lose morale and stop working. In this example, the selection process had an indirect effect on retention, mediated by its effect on programme legitimacy. In terms of performance, one study reported that CHW compliance with guidelines was lower when women in the village had an influence on selection compared to when they did not, although the results were not statistically significant [22].

The move toward payment of salaries and the recognition that CHWs may come from outside the area they serve (“trusted outsiders” [24]) in integrated/comprehensive programmes have led to formal human resource management of CHWs [25], including the introduction of “hybrid” approaches to selection in some areas. In Zambia, for example, potential CHWs required community support in order to apply, but final selection was undertaken centrally [14]. In this programme, there was very high competition for CHW posts, with an applicant to post ratio of 7.4:1 [14].

Selection processes for healthcare students and professionals have been studied in high income countries, with some methods of selection such as multiple mini interviews and situational judgment tests having good predictive validity for future performance, but other methods such as personal statements and unstructured interviews have been found to be less valid and thus less (if at all) useful. [26, 27] However there is an evidence gap in low income countries and the need for research into CHW selection in particular has been highlighted [14]. For example, Ballard et al. [12] note that “while the importance of appropriate CHW selection is repeatedly cited by narrative reviews as a precursor to success, uncertainly remains about how best to operationalize the process” (p. 11). The evidence base is also limited by poor descriptions of CHW selection processes [7, 28].

There is only one existing study which explicitly sought to evaluate the effectiveness of different selection criteria for CHWs, with CHW performance was assessed in three domains: role development, community health development and performance of skilled tasks [13]. The study results identified statistically significant positive relationships between selection scores and CHW performance for the selection criterion of volunteering spirit across all three domains, and the criteria of permanent residency and being respected within the community in two domains, but the study would be difficult to replicate because the methods used to assess each selection and performance criterion are poorly described. Kok and colleagues [7] identified nine studies that referred to (not always explicitly evaluating) the influence of community selection on CHW performance and, in contrast, 51 studies on the influence of incentives on CHW performance. Similarly, a review of CHW training in sub-Saharan Africa and Asia selected and reviewed over 100 articles and reports [29], highlighting the relative lack of work on CHW selection. Both incentives and training are also critical influences on CHW programme effectiveness and are related to selection: a selection process should be able to identify those candidates who will be most motivated by the incentives offered, as well as those candidates who are most “trainable” as CHWs. Thus the effect of incentives or training on CHW (programme) performance will, in part, be determined by the selection process employed, so selection needs to be considered (or at least fully described) as part of the “causal chain” in any study of CHW programme design features “downstream” of selection. Three further studies are worth including given the paucity of the evidence base in relation to CHW selection. The first [14] relates to the effect of CHW *recruitment* strategies on CHW performance and retention. CHWs were recruited using adverts that focused on either the “social mission” of CHW work, or the “career mission” of being a CHW. Over the 18 month follow-up period, no differences in CHW performance or retention were identified between the two groups. The second [30] evaluated the effect of various *socio-demographic characteristics* on CHW performance. The results suggest that CHWs aged between 30 and 40 and those with higher levels of education generally had the highest job performance; the authors hypothesise that CHWs in this age range are “energetic and socially settled”. The third [31] describes how CHW programme managers in the Kitgum district of Uganda decided to start giving preference to female applicants to provide better gender balance across the CHW cohort as a whole. The use of socio-demographics as part of any selection process would need to be considered very carefully however given the potential for discrimination.

Given the paucity of the current evidence base, studies of the effectiveness of existing selection processes for CHWs are urgently required. While different evaluation criteria can be applied [32], we focus on predictive validity in this paper. Predictive validity measures the extent to which performance in the selection process determines future performance in post [33].

## Methods

### Aim

The aim of this study is to evaluate the predictive validity of the existing selection tools for CHWs being used by *Living Goods* in Kenya.

### Study design

A cohort study of CHWs employed by *Living Goods* in eight local areas of Kenya was undertaken.

### Setting

The Kenyan Community Health Strategy 2014–19 [34] proposes five salaried Community Health Extension Workers and ten volunteer CHWs for every 5000 people (see McCollum et al. [35] for further details). This change sees more Extension Workers, who will need to have at least a basic certificate in social or community-related studies, and fewer CHWs per capita. The CHWs to retain their roles would had been selected using the guidelines that ran alongside the 2005–10 Community Health Strategy, with community-led selection criteria including the ability to read and write, being “concerned about the welfare of the people” and having “demonstrated attributes valued by the community” [36], p. 18. However it is not clear exactly how a community would determine which potential CHWs best fulfilled these criteria (i.e. no specific selection tools are proposed).

Several non-government organisations work in collaboration with the Kenyan Ministry of Health to provide local CHW programmes. *Living Goods* is one such organisation and has been operating in Kenya for three years. It currently operates in two counties where 1300 CHWs are deployed. There are plans to increase the number of *Living Goods* CHWs to 2700 across six counties by the end of 2020. Each CHW working with *Living Goods* is assigned approximately 100 households, but this figure varies across CHWs according to population density and therefore the time needed to travel between households. CHWs have responsibility for case management of malaria, diarrhea, pneumonia, pregnancy and newborn care, family planning and under-nutrition. *Living Goods* CHWs earn a small, motivating income from the sales of products and performance-based health incentives. CHWs are equipped with a smart Android phone which includes an app that helps them accurately diagnose and manage childhood illnesses, register and support pregnant women, and follow up with clients as well as collate data required by the Ministry of Health. These data also provide the on-the-job performance measures used in this study, which are described in more detail below. Previous interrogation of the performance data did not suggest under-performance of *Living Goods* CHWs; the motivation for this study was not therefore to seek explanations for, and address, reasons for poor performance. Instead we sought to determine the relationship between selection scores and on-the-job performance in a new setting. If a strong relationship existed, then the *Living Goods* tools could potentially be used in other settings following local adaptation. If it did not, then there would be scope for improving the selection process and potentially increasing on-the-job performance as CHWs selected using the new process are recruited and trained: an idea not dissimilar to that of Continuous Quality Improvement in healthcare [37].

### Selection process for CHWs at *Living Goods*

Community leaders, with input from community members, nominate potential *Living Goods* CHWs who meet the basic entry criteria (literacy, respected by the community, resident in the local area, willingness to volunteer and being aged over 18 years). Following nomination, potential CHWs are invited to participate in a selection event. These are hosted at local venues, such as health facilities. There are two stages to the selection process. First, candidates complete a short written test using pencil and paper, and are allowed around 10 min to complete this. There are three sections to the test: Business maths, where candidates are asked to write down their answers to four calculation questions; Comprehension, where candidates are asked to read a brief passage in English and answer three multiple choice questions; and About you, where candidates are asked five questions to evaluate motivation and honesty. Each section is worth a total of 10 marks. The total score for each section is entered into a mobile phone app by a member of the *Living Goods* recruitment team. The second stage involves a one-to-one interview with a member of the recruitment team. If candidates scored poorly on the Business maths section of the written test, they are asked to attempt these questions again to determine if it was the test environment which contributed to their poor score, rather than their lack of understanding. Candidates are then asked questions in eight key areas about their motivation for the CHW role (scored out of 30) and their ability to sell (scored out of 10). Example questions include involvement with local committees and ability to attend a two-week full-time training course around existing commitments. Each area is marked on a five point scale (1 = bad to 5 = excellent). The interviewer also notes any issues regarding the suitability of the candidate to perform the role of a CHW; for example if the candidate was heavily pregnant their application would be deferred for the time being. There is no fixed passing score for any element of the selection process; a holistic judgement regarding suitability is made by the recruitment team. The selection tools can be downloaded from https://chwimpact.org/appendices (Appendix B).

### Training and initial assessment of CHWs

Selected CHWs attend a two-week full-time training programme held at a local venue where they follow a structured curriculum. At the end of the training programme CHWs sit a written test, undertake a clinical examination of a child and complete a practical assessment during which they are asked to register a new household for CHW services, undertake a pregnancy registration and conduct a simulated post-natal follow-up. The pass mark for the assessment is 75%.

### On-the-job performance monitoring

CHWs record all of their health-related activities on the *Living Goods* app, and the data are automatically sent through to the Living Goods server. Although the data are evaluated at CHW-level and used as part of discussions between CHWs and their supervisors, they are not currently used for formal performance management of individual CHWs. Data are informally verified by supervisors and there is also a central quality control process for checking the accuracy of the data recorded by CHWs. CHWs’ sales are monitored as they visit the local office to restock goods they have sold (CHWs are required to buy the goods from the office and then sell them on for a small margin to their clients).

### Data

Data on all CHWs who graduated from their CHW training between April and December 2016 and who remained in post for at least 6 months were obtained.

The following demographic data for each CHW were obtained (Table 1):

- Local branch/area of work (8 branches included).
- Whether or not the CHW worked in a malaria-endemic area (binary).
- Gender (binary).
- Age (continuous). Three CHWs had recorded ages of under 20 (5, 10, and 14). These were re-coded as missing data.
- Whether or not the CHW had previously worked as a CHW before joining *Living Goods* (binary).
- Level of education achieved (categorical: Some primary, completed primary, some secondary, completed secondary, higher than secondary).

**Table 1:** CHW demographics (*N* = 547)

	*N* (%)^a^	Mean (SD), range
Branch:		
A (Malaria endemic)	31 (5.7%)	
B (Malaria endemic)	71 (13.0%)	
C	68 (12.4%)	
D (Malaria endemic)	70 (12.8%)	
E (Malaria endemic)	71 (13.0%)	
F (Malaria endemic)	97 (17.7%)	
G	124 (22.7%)	
H	15 (2.7%)	
Work in malaria endemic area total	340 (62.2%)	
Gender:		
Female	358 (65.5%)	
CHW before joining *Living Goods:*		
Yes	488 (89.2%)	
Level of education (*N* = 546):		
Some primary	449 (82.2%)	
Completed primary	11 (2.0%)	
Some secondary	28 (5.1%)	
Completed secondary	29 (5.3%)	
Higher than secondary	29 (5.3%)	
Age in years (*N* = 544):		41.1 (8.9), 23 to 70

^a^Percentages may not sum to 100% due to rounding

About two-thirds of the CHWs were female, most (just under 90%) had previously worked as a CHW and most (just over 80%) had “some primary” education. The mean age of CHWs was 41 years, but there was a wide range, from 23 to 70 years.

The score obtained on each part of the selection process was obtained. These are:

- Written test: Comprehension (possible range 0–10).
- Written test: Business maths (possible range 0–10).
- Written test: About you (possible range 0–10).
- Interview: Selling ability (possible range 0–10). Data were missing for 3 CHWs.
- Interview: Motivation (possible range 0–30). Data were missing for 3 CHWs.

The score obtained on each part of the post-training assessment was obtained. These are:

- Written test score (possible range 0–25).
- Clinical examination score (possible range 0–40).
- Practical assessment score (possible range 0–35).

There were positive Kendall's tau-b correlation coefficients between the three test components (written and clinical tau-b = 0.19; written and practical tau-b = 0.22; clinical and practical tau-b = 0.16; all *p* < 0.01), so to avoid over-testing, the three scores were combined into a single variable with possible range 0–100.

Four measures of on-the-job performance were used in the analysis, each summing performance across months 4–6 in post were obtained. These are:

- Total number of household and pregnancy registrations.
- Total number of under-1 and under-5 child assessments.
- Proportion of follow-ups required that were done on time (for CHWs who had at least one follow-up required; *N* = 52 CHWs had no follow-ups required). This variable was calculated from data on the number of follow-ups done on time (numerator) and the number of follow-ups required (denominator).
- Total net value of sales (sales – value of goods returned to the office). Because net sales figures were provided, this value could be negative (and was negative for *N* = 7 CHWs).

The data were supplied by *Living Goods* in a single Excel 2013 file. Each CHW was given a code so that all data were anonymous. Data were transferred into Stata v.14 for analysis [38].

### Methods of analysis

The aim of the analysis was to determine the predictive validity of each of the selection scores on post-training assessment score and on-the-job performance. To describe the data and determine which statistical tests should be employed, histograms of continuous variables and summary statistics for all variables were produced. We used medians and inter-quartile ranges (IQR) to describe the average and spread of selection scores, post-training assessment scores and on-the-job performance variables because, on visual inspection of the histograms, we found that some variables were highly skewed. We also evaluated whether CHWs’ sales activity could be “crowding out” their health activity, by calculating the Kendall’s tau-b correlation coefficients between sales and the three health activity outcomes.

Because of the skewed distributions of many of the variables and the number of ties, Kendall’s tau-b correlation coefficients were calculated using the split-ties method. The critical value for statistical significance was set at *p* < 0.01. The practical significance of the correlation coefficients was interpreted using the thresholds given by Cooper et al. [39]: Poor < 0.3, Adequate 0.3 to 0.39, Good 0.4 to 0.49 and Excellent > = 0.5. A sample size of 184 CHWs would be required to detect a correlation of at least 0.3 with 95% power and a two-tailed alpha of 0.01.

## Results

### Summary statistics

Five hundred fourty-eight CHWs were included in the study; one CHW was excluded from the sample as no selection scores were available. Summary statistics for selection scores, post-training assessment scores and on-the-job performance are shown in Table 2. While most CHWs in post achieved at least 50% of the marks available on all of the selection tests, there were some CHWs who scored no marks on four of the five components. There were positive correlations between the three written component scores and between the two interview component scores (all tau-b *r* > 0.30 with *p* < 0.01). There was a very small, not statistically significant negative correlation (tau-b = -0.04, *p*=0.25, *p* = 0.02) between total scores in the written and interview components.

**Table 2:** Selection scores, post-training assessment scores and on-the-job performance

	N (%)^a^	Median (IQR), range
Selection scores:		
Written comprehension/10		7 (4 to 10), 0 to 10
Written business maths/10		8 (4 to 10), 0 to 10
Written about you/10		8 (6 to 10), 0 to 10
Interview selling/10 (*N* = 544)		7 (6 to 8), 0 to 10
Interview motivation/30 (*N* = 544)		24 (22 to 24), 10 to 30
Post-training assessment scores:		
Written/25		21 (18 to 23), 1 to 25
Clinical/40		33 (30 to 37), 4 to 40
Practical/35		28 (25 to 32), 0 to 35
Total/100		82 (74 to 88), 41 to 100
On-the-job performance data:		
Total household and pregnancy registrations		10 (5 to 23), 0 to 171
Total under-1 and under-5 child assessments		62 (23 to 84), 0 to 226
On time follow-ups as a percentage of those required (*N* = 495)		67 (20 to 90), 0 to 100
Sales value (KSh)		2338 (643 to 6225), − 1100 to 60351
CHW (in)activity:		
CHWs with no household or pregnancy registrations in 3 months	32 (5.9%)	
CHWs with no under-1 or under-5 child assessments in 3 months	78 (14.3%)	
CHWs with no on-time follow-ups in 3 months (of those with at least 1 required)	106 (21.4%)	
CHWs with zero sales in 3 months	41 (7.5%)	
CHWs with no health activity and zero sales in 3 months	11 (2.0%)	

^a^Percentages may not sum to 100% due to rounding

There was also a wide range of post-training assessment scores and on-the-job performance, with all indicators for the latter having positively skewed distributions. Almost all CHWs (*N* = 515, 94%) had registered at least one household or pregnancy during months 4–6 in post and 469 (86%) had undertaken at least one child assessment. The median proportion of required follow-ups completed on time, across those CHWs with at least one follow-up required (*N* = 495), was 67%. However 106 of these CHWs (21%) had not undertaken any on-time follow-ups. 41 (7.5%) CHWs had no sales, but most of these (*N* = 30, 73%) had undertaken at least some health-related activity. Thus 11 CHWs (2%) had not undertaken any health-related activity *and* also had no sales in the period and may be inactive CHWs.

All three correlations between sales and the health activity areas were positive and statistically significant at *p* < 0.001, although fairly low (tau-b values between 0.12 and 0.18), suggesting that CHWs with higher health activity generally had higher sales and therefore that crowding out was not a problem.

### The relationship between selection scores and post-training assessment scores and on-the-job performance

**Fig. 1:**
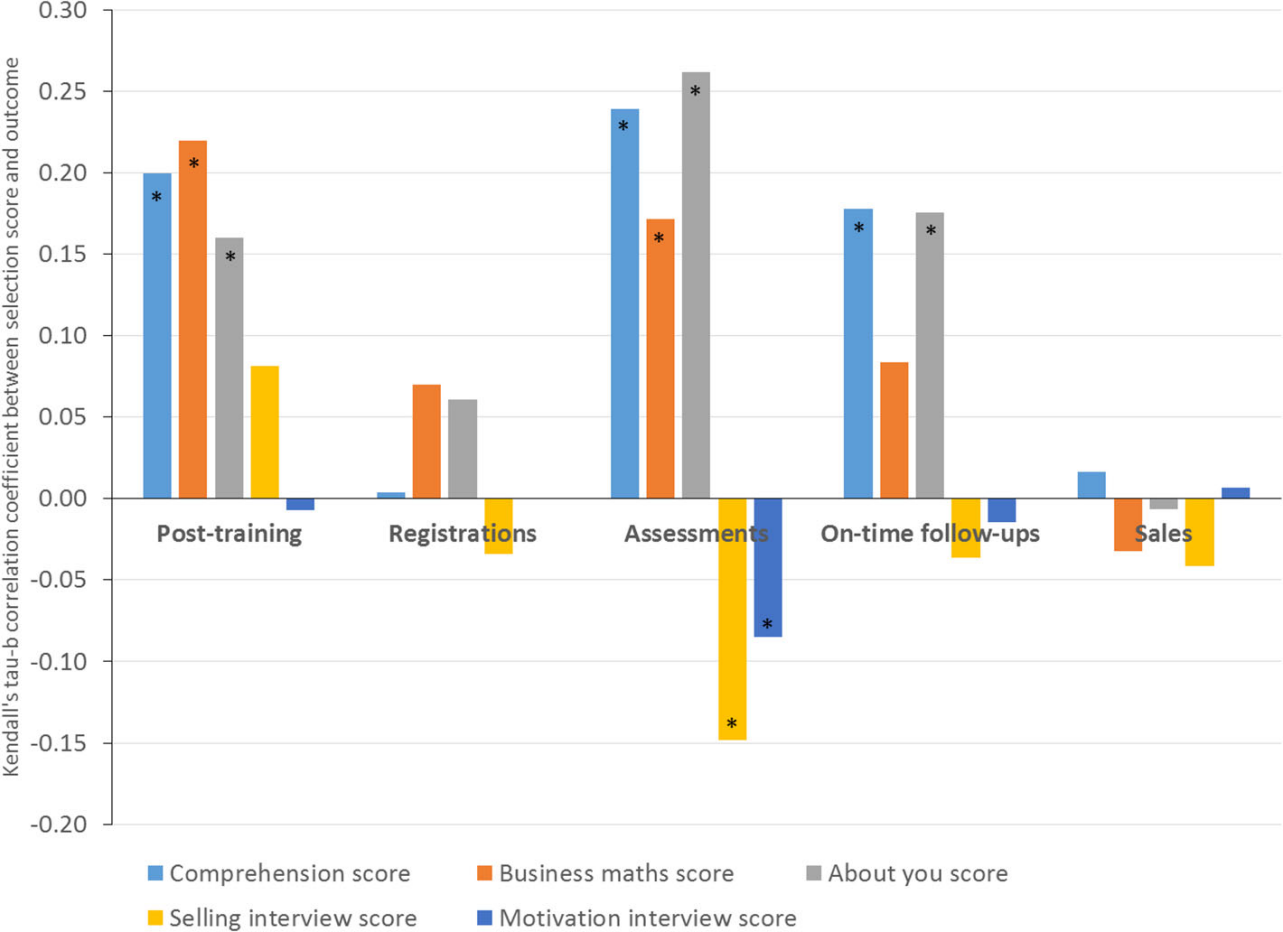
Predictive validity of selection scores. Coefficients that are statistically significant at *p* < 0.01 are indicated with a *

The Kendall’s tau-b correlation coefficients between each selection score and each outcome are shown in Fig. 1. All three elements of the written test are generally predictive of post-training assessment scores and on-the-job health-related performance, but none of the correlation coefficients reached 0.3, the level considered the minimum for an “adequate” selection test. There is some evidence that selection interview scores are negatively correlated with on-the-job health-related performance, particularly for the total number of child assessments undertaken.

## Discussion

The aim of this study was to evaluate the predictive validity of the existing selection tools for CHWs being used by *Living Goods* in Kenya. To do so, we compared the correlation coefficients estimated from the data used in this study with Cooper et al. [39]‘s thresholds: Poor < 0.3, Adequate 0.3 to 0.39, Good 0.4 to 0.49 and Excellent > = 0.5. We generally found statistically significant positive correlations between scores on the three written components of the selection process and post-training assessment scores and health-related on-the-job performance. However none of the correlations would be considered “adequate” using the 0.3 threshold. On the whole, interview scores were negatively correlated with health-related on-the-job performance. None of the components of the selection process were predictive of sales.

Although the importance of community involvement in selection is clearly acknowledged [18], the existing evidence does not consistently show that doing so improves the quality or retention of those selected for CHW roles or that the process is fair [13, 21–23]. Formal selection processes *may* provide an effective complement to community involvement, as there is evidence from a large meta-analysis that some methods of selection such as structured interviews are generally predictive of on-the-job performance [40] although the evidence base is dominated by studies undertaken in high income countries. Many CHW programmes already use selection tools. Because the tools used vary across contexts (and different tools may be differentially effective in different contexts), it is not possible to immediately generalise our findings to tools used by other programmes. However if they are not sufficiently valid in the context for which they were designed, they are unlikely to be valid elsewhere. Instead, our work highlights a need for work to evaluate existing tools to ensure they are actually helping CHW programme providers to achieve their health-related programme goals and provides a method for doing so. The lack of evidence for CHW programmes was one rationale for the production of a document entitled “Practitioner Expertise to optimize community health systems” [12], in which the *Living Goods* selection tools are cited as exemplars. However our work suggests that care is needed should others decide to use these existing tools which may not turn out to be sufficiently valid.

Even where other evidence exists [13], it is difficult to compare results on predictive validity because different selection criteria and performance outcomes were applied. When a greater volume of literature is available it may be possible to group similar selection criteria and performance outcomes to enable exploratory evidence synthesis.

Our sample size of over 500 CHWs meant we had sufficient statistical power to detect a correlation between selection scores and performance outcomes of at least 0.3 with a low alpha value (type I error). This meant we were able to evaluate the effect of the individual components of the selection process separately to provide evidence that may help other CHW programme providers develop effective and efficient selection processes.

As we lacked data for the candidates that were not selected for CHW roles we were unable to correct for restriction in range, although the selection scores of those actually selected covered the full range of those possible for four of the five components of the selection process. It would also have been helpful to have CHWs’ scores on each question in each test rather than just total scores. Such scores would have enabled us to calculate the internal consistency (reliability) of the selection tests and hence estimate the likelihood of a CHW achieving the same score had a different set of questions been used. This is important because a test can only be valid if it can consistently measure the construct of interest (i.e. if it is reliable). Likewise, we did not have selection data for CHWs who were not retained for at least 6 months, so we could not determine whether selection scores were predictive of retention in post. Studying retention is important because many CHW programmes suffer from relatively high attrition rates [18]. Two of the on-the-job performance outcomes, household and pregnancy registrations and child assessments, may reflect the opportunities available to CHWs to undertake these activities, rather than their motivation and ability to do so. The opportunity to undertake these activities will be affected by the number of households to which a CHW is assigned (which is determined by population density), the number of new or previously unregistered households (determined by previous CHW activity and migration; all CHWs would have had the opportunity to register at least one household however), and the average number of children in a household.

The data we did have on health-related on-the-job outcomes was based on that recorded by the CHWs themselves using their mobile phones. While some of these data are verified by supervisors, it would be impossible to verify it all. Nevertheless, there are no incentives for CHWs to over-report their activity and we had some CHWs in the sample who reported no activity at all, so there is no obvious reason to dispute CHWs’ honesty.

## Conclusion

None of the selection tests used by *Living Goods* for selection of their CHWs in Kenya were found to be adequate predictors of post-training assessment scores or the on-the-job performance indicators included in this analysis. It is worth reviewing the interview questions to determine if these need to be amended given the poor relationships between interview scores and performance outcomes. The written tests showed the most promise as potential predictors of performance; as the individual tests were short and therefore unlikely to be very reliable, it may be worth developing and evaluating a longer test to use as part of the selection process. Such changes may be particularly fruitful given *Living Goods’* plan to double the number of their CHWs working in Kenya in the next two years. As none of the performance outcomes used in this study directly assessed the *quality* of care provided by CHWs in the field or health outcomes; further study to do so and therefore to determine if selection scores are correlated with quality of care and health outcomes is therefore warranted. Other CHW programme providers should consider evaluating their own selection tools, ideally in collaboration with research teams, to ensure that the tools are contributing positively to the achievement of programme aims.

## Abbreviations

CHW: Community Health Worker
